# Positive effects on hematological and biochemical imbalances in patients with metastatic breast cancer stage IV, of BP-C1, a new anticancer substance

**DOI:** 10.2147/DDDT.S80451

**Published:** 2015-03-13

**Authors:** Steen Lindkær-Jensen, Stig Larsen, Nina Habib-Lindkær-Jensen, Hans E Fagertun

**Affiliations:** 1 Department of Surgery and Cancer, Hammersmith Hospital Campus, Imperial College, London, UK; 2 Center of Epidemiology and Biostatistics, Faculty of Veterinary Medicine, University of Life Science, Oslo, Norway; 3 Meddoc Research AS, Skjetten, Norway

**Keywords:** hemoglobin, hematocrit, neutrophils, thrombocytes, albumin, electrolytes

## Abstract

A benzene-poly-carboxylic acid complex with cis-diammineplatinum(II) dihydrocholride, BP-C1 is currently used in clinical trials in treating metastatic breast cancer. BP-C1 controls tumor growth with a few mild side-effects, improving quality of life.

**Methods:**

The data consisted of prospectively collected laboratory results from 47 patients in two controlled clinical trials of daily intramuscular injections of BP-C1 for 32 days. Study I was performed as an open, nonrandomized, Phase I dose–response, multicenter study with a three-level, between-patient, response surface pathway design. The second study was a randomized, double-blind, and placebo-controlled, multicenter study with a stratified semi-crossover design.

**Results:**

Hemoglobin (Hb) and hematocrit (Hct) increased significantly (*P*<0.01) during BP-C1 treatment, while red blood cell (RBC) count increased but not significantly. The most pronounced increase in Hb, RBC, Hct, and white blood cell (WBC) was in anemic patients (*P*≤0.01). WBC count and neutrophils increased significantly (*P*=0.01) in the overall data. WBCs and neutrophils (*P*<0.01), eosinophils (*P*=0.05) and monocytes (*P*<0.01) increased significantly and markedly in patients with lowest baseline levels. Additionally, low levels of thrombocytes significantly increased. No changes in liver parameters, amylase, glucose, creatinine, or albumin, were detected except for albumin in the subgroup with low baseline levels, where levels increased significantly (*P*=0.04). An increase in K^+^, Ca^2+^, and PO_4_^3−^ was most pronounced in patients with low baseline levels (*P*≤0.02). A similar pattern detected for Mg^2+^, prothrombin time (PT), coagulation factors II, VII, X (KFNT), and C-reactive protein (CRP), which increased significantly (*P*≤0.05) in the groups with the lowest values.

**Conclusion:**

Our findings support the safety profile of BP-C1 use in cancer patients. BP-C1 did not induce anemia, infection, bleeding, hepatic insufficiency or electrolyte imbalances. In contrast, BP-C1 corrected abnormalities. No hematological and biochemical toxicity was observed.

## Introduction

Low blood cell count is a known complication during cancer treatment.^1^,^2^ The European Cancer Anemia Survey (ECAS) evaluated the prevalence, incidence, and treatment of anemia in cancer patients.^3^ Data from 15,367 patients included tumor type, performance status, hemoglobin (Hb) levels, and cancer treatments. Prevalence of anemia at enrollment was 39.3% and was 67.0% during the survey, while the incidence of anemia was 53.7%, of which, 38.9% were treated. Anemia significantly correlates with poor performance status, which is compounded by the fact that many anemic patients remain untreated for their anemia. There are different causes of anemia amongst cancer patients: a prior low Hb level before the development of cancer, the cancer itself, and cancer treatments, such as radiotherapy and, more significantly, platinum-based chemotherapy. Anemia has been shown to be a useful biomarker in patients treated with rituximab-CHOP (combining cyclophosphamide, hydroxydaunorubicin, oncovin, and prednisone or prednisolone) immunotherapy.^4^

About 33% of cancer patients experience an infection as a result of chemotherapy, of which 57% are associated with neutropenia. This suggests that the risk of neutropenia has a significant impact on patients’ clinical care and quality of life (QoL). Neutropenia is a common and potentially life-threatening side effect of some chemotherapeutic regimens, due to the heightened risk of infection.^5^,^6^

Thrombocytopenia can be caused by bone marrow suppression, as a side effect of chemotherapeutic drugs or as a direct consequence of cancer (ie, infiltration of the bone marrow, which results in impaired production of platelets). Thrombocytopenia can be a serious complication.^7^ Within a cohort of 609 patients with solid tumors or lymphoma, bleeding occurred during 9% of cycles among patients with previous bleeding episodes (*P*<0.0001). Major bleeding episodes resulted in shorter survival and higher resource utilization (*P*<0.0001). Overall, the incidence of bleeding is low among patients with solid tumor; however, within some subgroups, the incidence exceeds 20%. Poor response to platelet transfusion is a clinically and financially significant downstream effect of thrombocytopenia.

The liver has a key role in the metabolism of many commonly used anticancer agents.^8^ Thus liver function assessment is a fundamental part of initial and ongoing management of patients with cancer. Chemotherapy in the setting of liver dysfunction can be associated with reduced effectiveness. Furthermore, several chemotherapy agents induce liver injury or dysfunction, which can manifest as abnormal serum liver biochemistry. Conventional serum liver biochemical testing does not always predict these potential complications. In the present study, we focused on current standard serum liver biochemical testing, measurements of liver physiology and pathophysiology, because of their limitations for chemotherapy dosing. This study highlighted the effect of treatment with benzene-poly-carboxylic acid complex with cis-diammineplatinum(II) (BP-C1) on serum liver biochemistry, in patients with stage IV metastatic breast cancer (MBC).

Increasing evidence links electrolyte disorders with greater morbidity and mortality, in critically ill patients. There are very few guidelines on the treatment of electrolyte disorders in critically ill patients,^8^,^9^ and most focus on individual electrolytes without taking the interrelation between specific deficits into account. Hyperkalemia, hypercalcemia, and hyponatremia can be chemotherapy-induced via various factors, for example, tumor lysis syndrome can cause hyperkalemia. Hypercalcemia and hyponatremia are often observed in patients with breast cancer, prostate cancer, and paraneoplastic syndrome. Hypercalcemia can also result from osteolysis but is mainly hormone-induced, such as in the case of parathyroid hormone-related protein. In some cases, hypomagnesemia results from drugs targeting epidermal growth factor receptors, such as cetuximab and panitumumab. Small-molecule targeted drugs, such as m-TOR inhibitors and ABL kinase inhibitors, can cause hypomagnesemia and hypophosphatemia. Thus, careful monitoring of the serum electrolyte concentration plays an important part in the biochemical monitoring of cancer patients and ensures effective and safe chemotherapy dosing in cancer, which is currently poorly defined. The purpose of our present investigation was to determine the effect of a new anticancer substance, BP-C1, which contains a very low content of cis-diammineplatinum(II) dichloride, on abnormal hematological and biochemical levels in stage IV MBC.^10^

## Methods

### Study design and participants

Prospectively collected laboratory results were obtained from 47 patients suffering from stage IV MBC, in two controlled clinical trials of daily intramuscular (IM) BP-C1 treatment for 32 days.^11^,^12^ Study 1 was performed as an open, nonrandomized, Phase I dose–response, multicenter study with a three-level, between-patient, response surface pathway (RSP) design.^13^ Of the 47 MBC patients, 15 were of Asian origin. According to the RSP design, five of the patients received a daily injection of 0.02 mg/kg BP-C1, one received 0.0256 mg/kg, one received 0.0275 mg/kg, one received 0.03 mg/kg, and four received 0.035 mg/kg. The mean age and body weight were 51.2 years (range 37–67 years) and 57.3 kg (range 45.3–78.0 kg) (Table 1). The patients had undergone from three to nine prior chemotherapy treatment cycles without known therapeutic effects. Study 2 was performed as a randomized, double-blind, and placebo-controlled, multicenter study, with a stratified semi-crossover design.^14^,^15^ The patient cohort consisted of 29 European and three Asian female MBC patients. All the patients received one daily IM injection of 0.035 mg/kg BP-C1 over 32 days. The mean age and body weight were 56.3 years (range 34.2–72.6) and 74.0 kg (range 44.0–101.0) (Table 1). All the included patients had previously undergone at least third-line chemotherapy and several other available types of cancer treatments.

### Procedures

Blood samples were taken at baseline (within 1 week prior to the BP-C1 treatment) and on the day after the end of BP-C1 treatment. Blood was drawn via a cubital vein and separated into serum by centrifugation. Standard procedures for measurement of the different parameters were used according to the standard methodology of the participating hospitals. All patients received one daily IM injection over 32 days. Approval of the ethical committees and the medical agencies in the participating countries were received, along with written consent from all patients.

### Statistical analysis

The results were expressed as mean value (standard deviation [SD]) and 95% confidence interval (CI) of the mean. For each of the observed variables, the quartiles were calculated. The 25Q, representing the upper limit of the first quartile (Q1) (the lower 25% of the observed data), and the 75Q, representing the lower limit of the fourth quartile (Q4) (the upper 25% of the observed data) were also calculated.

Changes from baseline to 32 days of treatment was calculated with paired Student’s *t*-test.^15^

The correlation coefficients (*r*) with 95% CI refer to Pearson linear correlation and the coefficients (*β0* and *β1*) in the linear regression analysis.^16^ Fisher z- transformation was used for testing and CI calculations.^16^ The increase from baseline to the end of BP-C1 treatment was correlated, and linear regression between the dependent increase during treatment and the independent baseline was performed.

### Outcomes

The study outcomes were hematological and biochemical values, recorded after 32 days of BP-C1 treatment.

## Results

### Hematology

**Figure 1:**
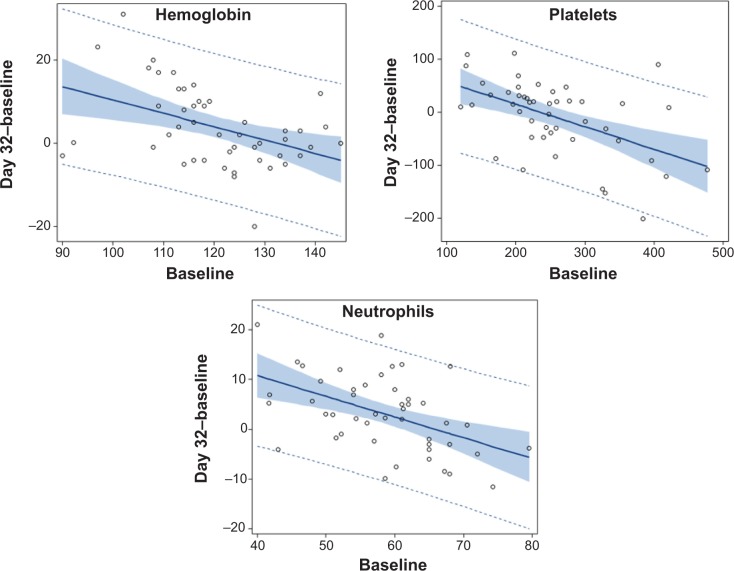
Hematology findings: regression between the increase from baseline to day 32 of BP-C1 treatment, and baseline. **Notes:** The full line represents the estimated regression line, the shaded area represents the 95% confidence interval of the regression line, and the dotted line represents the 95% prediction interval. The observed values are given as circles. **Abbreviation:** BP-C1, benzene-poly-carboxylic acid complex with cis-diammineplatinum(II).

Hb, white blood cell (WBC), and hematocrit (Hct) levels increased significantly (*P*<0.01) during the 32 days of treatment. Red blood cells (RBCs) increased during the same period but not significantly (Table 2). Mean cell volume (MCV) and ferritin levels were unchanged during treatment. The increase in Hb, WBC, Hct, and RBC was significantly (*P*<0.01) most pronounced among the patients in the Q1 subgroup, ie, with lowest baseline levels. No significant changes were detected among patients in the Q4 subgroup, ie, with highest baseline levels (Figure 1). Significant negative correlations (*P*<0.01) were detected between the increase during the 32 days of treatment, and the baseline, for Hb, RBC, WBC, and Hct (Table 3).

In the overall data, thrombocyte count was nearly unchanged during 32 days of BP-C1 treatment. However, a significant increase (*P*=0.02) was detected in the Q1 subgroup and a significant reduction (*P*=0.02) in the Q4 subgroup (Table 2). Significant negative correlations (*P*<0.01) were detected between the increase at day 32, and the baseline, for thrombocytes (Table 3). There was a significant increase in neutrophils (*P*=0.01), no significant increase in eosinophils, and a nearly unchanged basophil and monocyte count in the overall data (Table 2). In the subgroup Q1, significant increases in neutrophils (*P*<0.01), eosinophils (*P*=0.045), and monocytes (*P*<0.01) were detected. No significant changes were found in the Q4 subgroup (Figure 1). Significant negative correlations (*P*<0.01) were detected between the increase during the 32 days of BP-C1 treatment and the baseline level for neutrophils, eosinophils, and monocytes (Table 3). Lymphocyte levels decreased significantly during 32 days of BP-C1 treatment in the overall data. However, a borderline significant increase (*P*=0.08) was detected in the Q1 subgroup, while a significant decrease (*P*=0.01) was found in the Q4 subgroup, and a significant negative correlation (*P*<0.01) was found between increase in the lymphocytes during 32 days and the baseline level (Table 3).

### Serum chemistry

Gamma-glutamyltransferase (GGT), lactate dehydrogenase (LDH), and alkaline phosphatase (ALP) increased slightly but not significantly during 32 days of BP-C1 treatment in the overall data. A similar pattern was detected in both Q1 and Q4 (Table 4) subgroups. In the overall data, alanine aminotransferase (ALT) increased borderline significantly (*P*=0.08) during treatment. The increase in ALT was most pronounced and significant (*P*=0.04) among the Q1 patients. A nonsignificant reduction was detected in the Q4 subgroup, and a significant positive correlation (*P*<0.01) was detected between the increase during 32 days of treatment and ALT baseline (Table 3).

**Figure 2:**
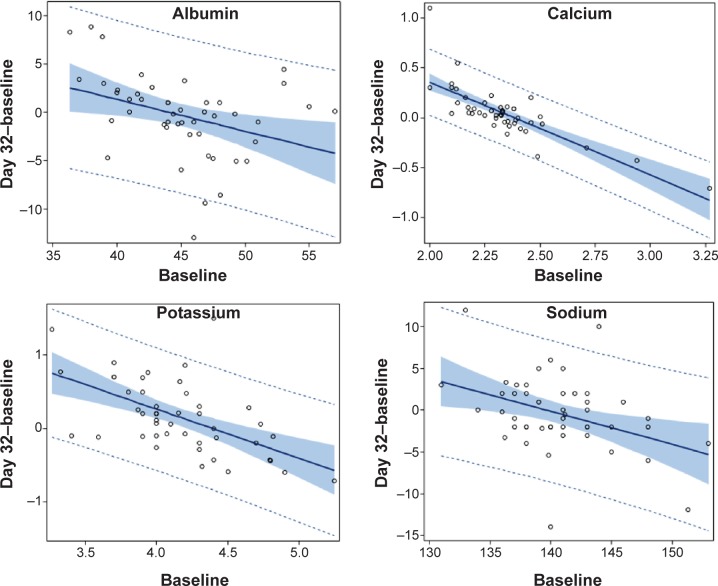
Clinical chemistry findings: regression between the increase from baseline to day 32 of BP-C1 treatment, and baseline. **Notes:** The full line represents the estimated regression line, the shaded area represents the 95% confidence interval of the regression line, and the dotted line represents the 95% prediction interval. The observed values are given as circles. **Abbreviation:** BP-C1, benzene-poly-carboxylic acid complex with cis-diammineplatinum(II).

In the overall data, no change in amylase and glucose was detected during the 32-day BP-C1 treatment (Table 4). A significant increase in amylase (*P*=0.04) was detected among patients in the Q1 subgroup, and a significant reduction (*P*=0.02) was found in the Q4 subgroup. Significant negative correlations (*P*<0.01) were detected between increase during 32 days treatment and the baseline amylase (Table 3). No significant changes in creatinine, albumin, or bilirubin were detected in the overall data. For albumin, the Q1 subgroup showed a significant increase (*P*=0.03) (Table 4). The Q1 subgroup analysis also detected a borderline significant increase in bilirubin (*P*=0.06), but no change in creatinine. No significant changes were detected for bilirubin and creatinine in the Q4 subgroup. The increase in albumin during BP-C1 treatment was significantly (*P*<0.01) negatively correlated to the baseline (Figure 2). K^+^, Ca^2+^, and PO_4_^3−^ increased significantly (*P*≤0.03) during treatment, in the overall data. The increases in K^+^, Ca^2+^, and PO_4_^3−^ were most pronounced among the patients in the Q1 subgroup (*P*≤0.01) (Table 4). In the Q4 subgroup, PO_4_^3−^ increased significantly (*P*=0.04), but no changes were detected for K^+^ and Ca^2+^ (Figure 2). No significant change in Mg^2+^ was detected in the overall data, but this increased significantly (*P*=0.01) in the Q1 subgroup. The increase in Mg^2+^ during treatment was significantly (*P*<0.01) negatively correlated to baseline (Table 3).

The prothrombin time (PT), KFNT, and C-reactive protein (CRP) were nearly unchanged during treatment in the overall data (Table 4). In the Q1 subgroup, PT, KFNT, and CRP increased significantly (*P*≤0.05), whereas KFNT was significantly reduced (*P*<0.01) among the Q4 patients. The increase in KFNT during treatment was significantly (*P*<0.01) negative correlated to baseline (Table 3). No such correlation pattern detected for PT or CRP.

## Discussion

A Phase I study demonstrated the clinical therapeutic benefit of BP-C1 in MBC,^11^ as did a Phase II randomized, double-blind, placebo-controlled, multicenter study with semi-crossover design.^12^ When 0.035 mg/kg BP-C1 was delivered intramuscularly for 32 days, it was found effective and also safe to use. Patients who completed 32 days of BP-C1 treatment were offered to continue BP-C1 (ie, open-label use) for an additional 32 days. In the Phase I study, 62.5% were classified as responders, with one patient a complete responder. The toxicity Bethesda NCT/CTC version 2.0^17^ increased in the low-dose group, but decreased in the high-dose group. In the Phase II study, the sum of target lesions increased by 2.4% in the BP-C1 group and by 14.3% in the placebo group. The increase in the placebo group was significant but was not in BP-C1. The difference between the groups was significant, in favor of BP-C1. There was a significant difference in favor of BP-C1 with respect to RECIST classification. The sum CTC-NCI toxicity score increased nonsignificantly in the BP-C1 group but increased significantly in the placebo group. The increase difference did not meet the level of significance. The sum toxicity score decreased in the patients receiving 64 days of BP-C1, from 9.2 at screening to 8.9 at day 48, but increased again to 10.1 and 10.6 during follow up. There was significant difference in favor of BP-C1. Patient responses of “Breast cancer treatment problem last week” was significantly decreased in the BP-C1 group and slightly increased in placebo. “Breast cancer related pain and discomfort” and “Physical activity problem” was significantly reduced during the 64 days of BP-C1 treatment. Thus these two clinical trials show a promising effect of BP-C1 for stage IV MBC patients.

We searched PubMed for reports published in English up to September 30, 2014, for chemotherapy agents preventing hamatological and biochemical disturbances as well as for nonchemotherapy agents to relieve side effects.^18^ We assessed all relevant articles for quality and relevance.^19^–^24^ Drug treatment requiring hospitalization or with known adverse effects and toxicity and no efficacy were not included in this systemic review, such as erythropoietin, platelet transfusion, electrolyte infusion or albumin infusion. It has been shown that primary prophylaxis with G-CSF in chemotherapy for epithelial ovarian cancer is of low significance.^19^ Docetaxel-loaded solid lipid nanoparticles (DSNs) have been developed to reduce systemic toxicity of docetaxel while still keeping its anticancer activity. Moreover, DSNs were shown to improve the main side effects of Taxotere^®^ by greatly lowering myelosuppression toxicity to bone marrow cells, in mice. These findings can be used to develop DSNs for human use.^20^ Treatment of cancer patients with herbo-mineral and metallic Ayurvedic drugs has been reported to significantly reduce the toxic effects of chemotherapy on various symptoms, and to have improved QoL,^21^,^22^ although no positive effects on hematological parameters could be demonstrated. Various herbal medicines have also been tested in cancer patients treated with chemotherapy, and these have been used to reduce chemotherapy-induced nausea and vomiting, to improve patients’ QoL and to allow their subsequent chemotherapy. But again, the effect of adjuvant herbs on hematological and/or biochemical imbalances were not tested.^23^,^24^ Results of the literature review suggest that there are a very limited amount of scientifically supported reports on the effect of innovative nontoxic drugs to alleviate chemotherapy- or cancer-induced toxicity with respect to hematological and/or biochemical imbalances (which potentially causes harm to our patients). Hopefully, drugs, such as BP-C1, will be developed to protect patients against cancer- and/or chemotherapy-induced toxicity in the future, thereby reducing serious cancer- and/or chemotherapy-induced complications, such as infections, anemia, bleedings, cachexia, and cardiovascular complications. In this context, BP-C1 seems to be an effective and a well-tolerated substance for patients with stage IV MBC, irrespective of previous chemotherapy treatment.

The promising effect of BP-C1 on patients with stage IV MBC is further substantiated in the present study, which clearly demonstrated that BP-C1 is superior to chemotherapy drugs concerning changes in hematology and biochemistry levels.^1^,^3^,^5^–^7^ The breaking news is that BP-C1 corrects these levels in MBC patients with low baseline values after chemotherapy. It is well known that chemotherapy drugs and some biological therapies can reduce patients’ RBC, WBC, and platelet counts substantially. Further, tumor lysis syndrome can occur secondary to chemotherapy, in which killed cancer cells are broken down by the body, causing hyperuricemia, hyperkalemia, hyperphosphatemia, and hypocalcemia.^18^ Shibata has recently underlined the importance of careful monitoring of serum Mg^2+^ and PO_4_^3−^ ions in patients treated with small-molecule targeted drugs, to which small attention has been paid so far.^25^

Levels of K^+^, Na^+^, and PO_4_^3−^ must be maintained within a very narrow margin to avoid arrhythmia and kidney failure. Furthermore, the majority of cancer patients undergoing chemotherapy develop anemia during their treatment. The ECAS, a study conducted across 24 nations in Europe, reported that about 83% of patients who received chemotherapy demonstrated anemia.^3^ Anemia often increases symptoms such as fatigue, weakness, and dyspnea; thus, it may worsen QoL and performance status in cancer patients. Anemia can also affect the prognosis of cancer patients, resulting in higher mortality. Chemotherapy-induced anemia in cancer patients is often underestimated and is not appropriately treated by health care providers. The ECAS showed that 53% of cancer patients did not receive any treatment for their anemia. BP-C1 seems to be the drug of choice to prevent anemia, neutropenia, and tumor lysis syndrome, thereby improving physical/nonphysical functioning, QoL, and prognosis in cancer patients. However, larger trials are necessary comparing BP-C1 with classical chemotherapy drugs on this matter.

Systemic infections are frequent and a serious complication in neutropenic patients.^26^ Preventing neutropenia is extremely important to achieve a successful outcome for patients undergoing chemotherapy. In contrast to most chemotherapy drugs, health care professionals can administer BP-C1 in the patients’ own home, and BP-C1 is not associated with neutropenia, thereby avoiding the need for human recombinant G-CSF that is used to treat neutropenia after chemotherapy.^27^,^28^ As stated by Hurvitz et al chemotherapy-related adverse effects in MBC are associated with a substantial economic burden for society, which increases with the number of adverse effects reported.^29^ With none or fewer complications associated with BP-C1 treatment, the financial impact may be eliminated. BP-C1 is not associated with low thrombocyte counts and was able to normalize the thrombocyte count after chemotherapy, in contrast to other anticancer drugs.^30^ Electrolyte imbalance correction, including that of serum albumin in patients with hypoalbuminemia, was observed after chemotherapy and BP-C1.

According to Kayl and Meyers, chemotherapy and its associated side effects can result in cognitive dysfunction with an adverse effect on QoL.^31^,^32^ In contrast, this has never been observed with BP-C1. In order to understand the mechanism of actions behind these surprising and extremely positive effects of BP-C1 on hematological and biochemical abnormalities in cancer patients, we are currently carrying out genetic studies on MBC patients.^33^

## Conclusion

BP-C1 seems to be the drug of choice to prevent anemia, neutropenia, tumor lysis syndrome, and biochemical imbalances, to improve physical as well as nonphysical function, QoL, and prognosis in cancer patients.

## Figures and Tables

**Table 1:** Demographic data

Factor	Study 1 (n=15)		Study 2 (n=32)	
	Mean (SD)	Min – max	Mean (SD)	Min – max
**Demographic factors and vital signs**				
Age (years)	51.2 (7.8)	37.0–67.2	56.3 (9.8)	34.2–72.6
BMI (kg/m^2^)	23.6 (3.2)	17.9–29.4	28.3 (5.2)	20.1–38.3
Disease duration (year)	5.4 (4.5)	1.8–18.0	6.6 (4.5)	1.1–19.0
Systolic BP (mmHg)	119 (12.6)	95–143	130 (10.9)	104–150
Diastolic BP (mmHg)	78 (9.7)	48–90	79 (8.1)	51–90
Heart rate (beat/min)	79 (10.8)	73–85	81.2 (9.0)	68–115
Respiratory rate (breath/min)	18 (2.3)	12–22	17.2 (1.8)	14–22
**Previous cancer treatment**				
Surgery	13/15		28/32	
Chemotherapy	15/15		32/32	
Hormone therapy	9/15		25/32	
Antibody therapy	0/15		4/32	
Radiotherapy	4/15		23/32	
Others	0/15		4/32	

**Abbreviations:** BMI, body mass index; BP, blood pressure; SD, standard deviation.

**Table 2:** Change in hematological values from baseline to day 32 of BP-C1 treatment, for the overall data and for patients in the lower quartile

	Overall data (n=47)		*P*-value	First quartile (n=12)		*P*-value
	Baseline	32 days		Baseline	32 days	
Hemoglobin (g/L)	120.4	124.2	*P*=0.01	105.5	117.0	*P*<0.01
	116.7–124.1	120.7–127.8		100.8–110.2	108.5–125.5	
Red blood cell count (10^6^/μL)	4.1	4.2	*P*=0.20	3.5	3.8	*P*<0.01
	3.9–4.3	4.1–4.3		3.2–3.8	3.5–4.1	
Hematocrit (%)	36.9	37.8	*P*=0.01	32.4	34.8	*P*=0.02
	35.8–37.9	36.8–38.9		30.7–34.2	32.2–35.5	
Mean cell volume (fL)	90.9	89.4	*P*=0.24	81.2	80.9	*P*=0.73
	88.1–93.8	86.7–92.1		75.5–87.0	74.9–86.8	
Platelet count (10^9^/L)	256.2	247.5	*P*=0.40	166.0	207.8	*P*=0.02
	231.5–280.8	225.0–270.0		145.7–186.3	168.3–247.4	
White blood cell count (10^9^/L)	5.82	6.51	*P*=0.01	3.70	5.96	*P*<0.01
	5.23–6.41	5.95–7.08		3.48–3.92	4.77–5.14	
Neutrophils (%)	58.3	61.5	*P*=0.01	46.7	53.9	*P*<0.01
	55.7–61.0	59.0–64.0		44.0–49.4	49.4–58.5	
Eosinophils (%)	2.37	2.90	*P*=0.16	0.41	1.86	*P*=0.05
	1.70–3.03	2.10–3.69		0.16–0.66	0.47–3.26	
Basophils (%)	0.33	0.34	*P*=0.86	0.00	0.12	*P*=0.10
	0.21–0.45	0.23–0.46		–*	0.00–0.27	
Monocytes (%)	7.61	6.95	*P*=0.12	5.06	6.61	*P*<0.01
	8.85–8.37	6.37–7.54		4.46–5.66	5.72–7.49	
Lymphocytes (%)	30.9	28.1	*P*=0.02	21.2	25.0	*P*=0.08
	28.5–33.3	25.7–30.4		18.8–23.7	19.8–30.2	

**Notes:** BP-C1, benzene-poly-carboxylic acid complex with cis-diammineplatinum(II);

* all values equal 0. The results are expressed as mean values and 95% confidence intervals.

**Table 3:** Correlation between increase during the 32-day treatment and baseline

	Hematology	Serum chemistry	
Hemoglobin	−0.43	ϒ-GT	0.16
	−0.64 to −0.16		−0.13 to 0.44
RBC	−0.57	LDH	0.40
	−0.73 to −0.33		0.12 to 0.62
Hematocrit	−0.36	ALP	−0.03
	−0.59 to −0.08		−0.31 to 0.26
MCV	−0.33	Amylase	−0.72
	−0.57 to −0.05		−0.84 to −0.55
Platelet count	−0.51	Glucose	−0.14
	−0.69 to −0.26		−0.42 to 0.15
WBC	−0.48	Creatinine	−0.29
	−0.67 to −0.22		−0.53 to −0.01
Neutrophils	−0.49	Albumin	−0.36
	−0.68 to −0.23		−0.59 to −0.08
Eosinophils	−0.36	Bilirubin	−0.21
	−0.59 to −0.08		−0.47 to 0.08
Basophiles	−0.47	Urea	0.28
	−0.67 to −0.21		−0.01 to 0.52
Monocytes	−0.73	Potassium	−0.56
	−0.84 to −0.56		−0.73 to −0.33
Lymphocytes	−0.49	Sodium	−0.39
	−0.68 to −0.23		−0.61 to −0.12
		Calcium	−0.72
			−0.88 to −0.65
		Magnesium	−0.51
			−0.70 to −0.25
		Phosphate	0.20
			−0.10 to 0.47
		PT	−0.72
			−0.83 to −0.54
		KFNT	−0.69
			−0.82 to −0.50
		CRP	0.05
			−0.26 to 0.35

**Note:** The results were expressed as the Pearson linear correlation coefficient with 95% confidence interval (using the Fisher z-transformation).

**Abbreviations:** ALP, alkaline phosphatase; CRP, C-reactive protein; LDH, lactate dehydrogenase; KFNT, coagulation factors II, VII, X; MCV, mean corpuscular volume; PT, prothrombin time; RBC, red blood cell; WBC, white blood cell; ϒ-GT, γ-glutamyl transferase.

**Table 4:** Change in serum chemical values from baseline to day 32 of BP-C1 treatment, for the total data and for patients in the lowers quartiles

	Overall data (n=47)		*P*-value	First quartile (n=12)		*P*-value
	Baseline	32 days		Baseline	32 days	
ϒ-GT (U/L)	58.3	159.3	0.11	17.0	45.6	0.16
	33.3–83.2	27.8–290.7		14.8–19.2	3.3–88.0	
LDH (U/L)	480.6	690.6	0.05	259.4	632.6	0.32
	317.4–643.9	373.4–1,007.7		240.0–278.7	0–1,415	
ALP (U/L)	192.9	327.7	0.22	77.0	86.8	0.11
	158.2–277.7	107.7–547.7		62.5–91.5	71.1–101.2	
ALT (U/L)	24.29	34.95	0.08	11.16	21.44	0.04
	19.27–29.31	20.35–49.56		10.20–12.12	11.61–31.27	
Amylase (U/L)	74.1	73.0	0.88	28.3	38.3	0.04
	54.3–93.9	59.4–86.6		24.1–32.5	28.0–48.7	
Glucose (mmol/L)	5.82	5.93	0.59	4.78	4.79	0.98
	5.43–6.20	5.41–6.45		4.54–5.02	4.38–5.19	
Creatinine (μmol/L)	73.4	73.9	0.83	52.3	54.5	0.60
	68.1–78.8	67.9–79.9		49.9–54.7	44.1–64.9	
Albumin (g/L)	45.1	44.7	0.57	39.3	42.1	0.04
	43.7–46.5	43.3–46.2		38.3–40.3	40.0–44.2	
Bilirubin (μmol/L)	10.1	13.0	0.45	6.0	7.7	0.06
	8.8–11.5	5.6–20.4		5.3–6.8	6.1–9.4	
Urea (mmol/L)	7.42	9.42	0.003	4.14	4.76	0.07
	6.29–8.55	7.59–11.34		3.74–4.55	3.95–5.56	
Potassium (mmol/L)	4.15	4.31	0.03	3.69	4.17	0.002
	4.03–4.28	4.19–4.44		3.55–3.83	3.91–4.42	
Sodium (mmol/L)	140.7	140.3	0.51	136.3	137.3	0.32
	139.4–142.0	138.8–141.7		135.2–137.4	135.5–139.0	
Calcium (mmol/L)	2.33	2.38	0.21	2.17	2.38	0.003
	2.25–2.40	2.33–2.43		2.12–2.21	2.75–2.48	
Magnesium (mmol/L)	1.15	1.12	0.42	0.77	0.82	0.01
	0.98–1.34	0.98–1.27		0.75–0.79	0.78–0.87	
Phosphate (mmol/L)	1.92	2.16	<0.001	0.92	1.15	0.004
	1.53–2.32	1.72–2.61		0.87–0.97	1.01–1.29	
PT	13.78	13.3	0.17	11.12	11.74	0.05
	13.00–14.55	12.75–13.85		10.66–11.59	10.87–12.61	
KFNT	1.00	0.97	0.10	0.89	0.94	0.02
	0.97–1.02	0.95–1.00		0.87–0.92	0.91–0.97	
CRP (mg/L)	5.06	7.49	0.34	0.89	2.92	0.008
	3.30–6.52	2.00–12.94		0.49–1.30	1.61–4.22	

**Note:** The results are expressed as mean values with 95% confidence intervals.

**Abbreviations:** ALP, alkaline phosphatase; ALT, alanine transaminase; BP-C1, benzene-poly-carboxylic acid complex with cis-diammineplatinum(II); CRP, C-reactive protein; LDH, lactate dehydrogenase; KFNT, coagulation factors II, VII, X; PT, prothrombin time; ϒ-GT, ϒ-glutamyl transferase.

## References

[1] Lynn JJ, Chen KF, Weng YM, Chiu TF (2013). Risk factors associated with complications in patients with chemotherapy-induced febrile neutropenia in emergency department. Hematol Oncol.

[2] Del Conte G, Sessa C, von Moos R (2014). Phase I study of olaparib in combination with liposomal doxorubicin in patients with advanced solid tumours. Br J Cancer.

[3] Ludwig H, Van Belle S, Barrett-Lee P (2004). The European Cancer Anaemia Survey (ECAS): a large, multinational, prospective survey defining the prevalence, incidence, and treatment of anaemia in cancer patients. Eur J Cancer.

[4] Hong J, Woo HS, Kim H (2014). Anemia as a useful biomarker in patients with diffuse large B-cell lymphoma treated with R-CHOP immunochemotherapy. Cancer Sci.

[5] Kuderer NM, Dale DC, Crawford J, Cosler LE, Lyman GH (2006). Mortality, morbidity, and cost associated with febrile neutropenia in adult cancer patients. Cancer.

[6] Shaikh AJ, Bawany SA, Masood N (2011). Incidence and impact of baseline electrolyte abnormalities in patients admitted with chemotherapy induced febrile neutropenia. J Cancer.

[7] Elting LS, Rubenstein EB, Martin CG (2001). Incidence, cost, and outcomes of bleeding and chemotherapy dose modification among solid tumor patients with chemotherapy-induced thrombocytopenia. J Clin Oncol.

[8] Field KM, Dow C, Michael M (2008). Part I: Liver function in oncology: biochemistry and beyond. Lancet Oncol.

[9] Geerse DA, Bindels AJ, Kuiper MA, Roos AN, Spronk PE, Schultz MJ (2010). Treatment of hypophosphatemia in the intensive care unit: a review. Crit Care.

[10] Fares F, Azzam N, Fares B, Larsen S, Lindkaer-Jensen S (2014). Benzene-poly-carboxylic acid complex, a novel anti-cancer agent induces apoptosis in human breast cancer cells. PLoS One.

[11] Dewi S, Larsen S, Srimuninnimit V, Lu YS, Manuaba T, Lindkær-Jensen (2013). Benzene-poly-carboxylic acids complex with cis-diammineplatinum (II) dichloride in the treatment of stage IV breast cancer patients. The Open Breast Cancer Journal.

[12] Larsen S, Butthongkomvong K, Manikhas A (2014). BP-C1 in the treatment of patients with stage IV breast cancer: a randomized, double-blind, placebo-controlled multicenter study and an additional open-label treatment phase. Breast Cancer.

[13] Dewi S, Kristiansen V, Lindkær-Jensen S, Larsen S (2014). Between- and within-patient n-level response surface pathway design in dose-finding studies. Open Access J Clin Trials.

[14] Carlsen KH, Kramer J, Fagertun HE, Larsen S (1993). Loratadine and terfenadine in perennial allergic rhinitis. Treatment of nonresponders to the one drug with the other drug. Allergy.

[15] Altman DG (1990). Practical Statistics for Medical Research.

[16] Anderson TW (1984). An Introduction to Multivariate Statistical Analysis.

[17] National Cancer Institute [webpage on the Internet] (1999). Cancer therapy evaluation program: common toxicity criteria manual.

[18] Davidson MB, Thakkar S, Hix JK, Bhandarkar ND, Wong A, Schreiber MJ (2004). Pathophysiology, clinical consequences, and treatment of tumor lysis syndrome. Am J Med.

[19] Matsui K, Mori T, Sawada M (2014). Evaluation of primary prophylaxis with granulocyte colony-stimulating factor for epithelial ovarian cancer. Eur J Gynaecol Oncol.

[20] Yuan Q, Han J, Cong W (2014). Docetaxel-loaded solid lipid nanoparticles suppress breast cancer cells growth with reduced myelosuppression toxicity. Int J Nanomedicine.

[21] Deshmukh V, Kulkarni A, Bhargava S (2014). Effectiveness of combinations of Ayurvedic drugs in alleviating drug toxicity and improving quality of life of cancer patients treated with chemotherapy. Support Care Cancer.

[22] Vyas P, Thakar AB, Baghel MS, Sisodia A, Deole Y (2010). Efficacy of Rasayana Avaleha as adjuvant to radiotherapy and chemotherapy in reducing adverse effects. Ayu.

[23] Goel HC, Prasad J, Singh S (2004). Radioprotective potential of an herbal extract of Tinospora cordifolia. J Radiat Res.

[24] Wang CZ, Calway T, Yuan CS (2012). Herbal medicines as adjuvants for cancer therapeutics. Am J Chin Med.

[25] Shibata H (2010). Cancer and electrolytes imbalance. Gan To Kagaku Ryoho.

[26] Penack O, Rempf P, Eisenblätter M (2007). Bloodstream infections in neutropenic patients: early detection of pathogens and directed antimicrobial therapy due to surveillance blood cultures. Ann Oncol.

[27] Miller RC, Steinbach A (2014). Growth factor use in medication-induced hematologic toxicity. J Pharm Pract.

[28] Bicakli DH, Varol U, Degirmenci M (2014). Adjuvant chemotherapy may contribute to an increased risk for metabolic syndrome in patients with breast cancer. J Oncol Pharm Pract.

[29] Hurvitz S, Guerin A, Brammer M (2014). Investigation of adverse-event-related costs for patients with metastatic breast cancer in a real-world setting. Oncologist.

[30] Chen A, Chen L, Al-Qaisi A (2015). Everolimus-induced hematologic changes in patients with metastatic breast cancer. Clin Breast Cancer.

[31] Hwang SY, Chang SJ, Park BW (2013). Does chemotherapy really affect the quality of life of women with breast cancer?. J Breast Cancer.

[32] Kayl AE, Meyers CA (2006). Side-effects of chemotherapy and quality of life in ovarian and breast cancer patients. Curr Opin Obstet Gynecol.

[33] Lamba JK, Fridley BL, Ghosh TM, Yu Q, Mehta G, Gupta P (2014). Genetic variation in platinating agent and taxane pathway genes as predictors of outcome and toxicity in advanced non-small-cell lung cancer. Pharmacogenomics.

